# Clinical reasoning – an approach for decision-making in education and training for biomedical scientists

**DOI:** 10.3205/zma001289

**Published:** 2019-11-15

**Authors:** Angelika Homberg, Heidi Oberhauser, Sylvia Kaap-Fröhlich

**Affiliations:** 1 Universitätsklinikum Heidelberg, Abteilung Allgemeinmedizin und Versorgungsforschung, Heidelberg, Germany; 2 fh gesundheit, Bachelor/Master Biomedizinische Analytik, Innsbruck, Austria; 3 Careum Stiftung, Bereich Bildungsentwicklung, Zürich, Switzerland

**Keywords:** Problem-solving, clinical decision-making, medical education, clinical competence, interprofessional relations, clinical laboratory personnel

## Abstract

**Aim:** Explicitly addressing clinical reasoning (CR) is seen as a promising opportunity in the teaching of the biomedical sciences to enable students to acquire the skills to meet the challenges posed by ever more complex health care processes. The quality of diagnostic decisions plays an essential role here. Our aim is to examine if biomedical scientists recognize the practical relevance of CR and are able to apply it as a reflective framework for their professional practice.

**Method:** In two different educational settings, biomedical science students were asked to look closely at CR in the context of the degree program and to indentify the different forms of reasoning used in their internships and professional practice. The written descriptions were analyzed for content and discussed in the seminars.

**Results: **In both scenarios, the analyses of the students’ descriptions and discussions showed that examining the different forms of CR helped to raise conscious awareness of thought and decision-making processes, encouraging students to think critically about them and to articulate insights about them, as well as recognize the importance of different reasoning strategies when making specific medical decisions.

**Conclusion: **CR for biomedical scientists could help make decision-making processes visible for other occupational groups and thus advantageously integrate specific professional expertise into health care.

Over the long term, an interdisciplinary focus on CR could foster and promote the development of a shared discourse and interprofessional collaboration.

## Zusammenfassung

**Zielsetzung: **Die explizite Auseinandersetzung mit Clinical Reasoning (CR) wird in der Ausbildung der Gesundheits- und Medizinalberufe als vielversprechende Möglichkeit gesehen, um für die Herausforderungen komplexer werdender Gesundheitsversorgungsprozesse zu qualifizieren. Die Qualität diagnostischer Entscheidungen spielt dabei eine elementare Rolle. Ziel dieses Projektes ist, zu überprüfen, ob Medizinisch-technische Laborassistenten/Biomedizinische Analytiker (MTLA/BMA) die praktische Relevanz des CR erkennen und als Reflexionsrahmen für ihr berufliches Handeln nutzen können.

**Methodik: **In zwei unterschiedlichen Bildungsszenarien setzen sich MTLA/BMA in Anlehnung an das Lernfeldkonzept mit CR auseinander und identifizieren unterschiedliche Reasoningformen in ihrem Praktikumseinsatz/beruflichen Alltag. Die schriftlichen Aufzeichnungen wurden inhaltsanalytisch ausgewertet und in den Seminaren mündlich diskutiert.

**Ergebnisse: **Die Auswertungen der Aufzeichnungen und die Diskussionen der Studierenden zeigten in beiden Szenarien, dass die Auseinandersetzung mit den unterschiedlichen CR-Formen dazu beitrug, Denk- und Entscheidungsprozesse ins Bewusstsein zu rufen, kritisch zu hinterfragen und sprachlich zum Ausdruck zu bringen sowie die Bedeutung unterschiedlicher Reasoning-Strategien in konkreten beruflichen Entscheidungsprozessen zu erkennen.

**Schlussfolgerung: **CR für MTLA/BMA könnte dazu beitragen, Entscheidungswege für andere Berufsgruppen sichtbar zu machen und auf diese Weise das professionsspezifische Wissen in Patientenversorgungsprozesse gewinnbringend zu integrieren.

Langfristig könnte die berufsgruppenübergreifende Auseinandersetzung mit CR die Entwicklung eines gemeinsamen Sprachduktus und interprofessionelle Zusammenarbeit fördern.

## 1. Introduction

Since health care decisions are becoming increasingly complex, there is a need to develop professional frameworks [1], [2] and for coordinated interprofessional collaboration among medical teams [3]. Concepts describing the decision-making process, such as clinical reasoning (CR), can assist such processes because the particular patterns of reasoning are made visible [4], [5], [6]. There are many definitions of CR [7], [8]. Generally, what is meant are the complex thinking processes engaged in by health care professionals, to which expertise, cognition and meta-cognition, and hypothetico-deductive approaches are central. Different forms of reasoning are categorized [6] in the literature with emphasis on various aspects such as ethics, interaction and relevance to science [7], [9], [10].

In Germany, CR in medical education has been more implicitly taught, even though the explicit embedding of CR in the curriculum appears very promising [11], [12], [13]. In the therapeutic occupations, such as physiotherapy and occupational therapy, a more direct grappling with CR has had a long tradition and has been anchored in the curriculum in many ways [7], [14], [15], [16], [17]. Forms of CR are described in the practice of laboratory diagnostics which, in addition to technical and scientific aspects, emphasize the logic of clinical and patient-centered decision-making [4], [18]. These are hardly covered in the education of biomedical scientists, although these occupations assume an elementary role in the process of providing health care. They are responsible for generating valid findings to serve as the basis for subsequent clinical decisions. In regard to diagnostic processes, it has been noted that the interactive use of reasoning concepts can reduce errors [19], [20] and encourage the integration of internal evidence in the decision-making process [21]. Oberhauser outlines in a theoretical fashion how individual forms of reasoning can be applied to the practice of the biomedical sciences [22].

## 2. Project description and method

The model of CR according to Higgs & Jones is covered in detail with students in two selected educational settings at different universities and involving different degree programs (see table 1 (Tab. 1)). The corresponding seminars specifically address scientific, conditional, interactive, narrative, pragmatic and ethical reasoning. The aim of these seminars is to foster and encourage critical examination of the students’ own decisions and their development of professional patterns of reasoning by applying theory to reflect on practical decisions.

To investigate the transferability of the CR concept to biomedical science, assignments were given based on the degree program [23] to elicit application of theoretical knowledge to real work situations.

Ten students enrolled in the bachelor degree program in Interprofessional Healthcare at the University of Heidelberg’s School of Medicine [24], [25] were asked to describe concrete examples of the pre-analytical, analytical and post-analytical work processes for the forms of reasoning listed above and to justify each decision that was made based on what knowledge was applied.

Independently of this, 37 students in the master degree program in Biomedical Sciences at the Health University of Applied Sciences (*fh gesundheit*) in Innsbruck and Berlin reflected on their practical work experiences in which forms of reasoning were used and differentiated between those they were most familiar with and those that needed to be worked on and refined. In both of the degree programs these questions were processed in writing and then discussed as a group. The written texts were analyzed for content [26] (see table 2 (Tab. 2)).

## 3. Results

The undergraduate students studying biomedical sciences in Heidelberg were able to find concrete examples of all seven forms of reasoning in the stages of pre-analysis, analysis, and post-analysis. In addition to professional expertise acquired through education, the students specifically identified the workplace’s Standard Operating Procedures (SOP), their own experiences, and the sharing of information among colleagues as comprising the body of knowledge on which their reasoning was based. In respect to ethical reasoning, the students also identified their own discretion as playing a role. Students did not feel themselves to be sufficiently qualified to engage in interactive or ethical reasoning.

The graduate students in Innsbruck and Berlin were also able to recognize all of the forms of reasoning within the analytical biomedical processes of their work. The most familiar forms of reasoning for them were scientific, interactive and pragmatic reasoning (see table 3 (Tab. 3)). It is striking that, in contrast to the undergraduate students, the graduate students reported that conditional, narrative and ethical reasoning were also among the most familiar forms to them. The practical relevance of CR was acknowledged in both degree programs, and the students were successful in applying the theoretical concept to their professional practice and in reflecting upon their own decision-making processes. Due to their professional experience, the graduate students provided considerably more complex descriptions of concrete work situations than the undergraduates, as was expected. Ethical and narrative reasoning in the context of the biomedical sciences was cited in both educational settings as needing further refinement.

## 4. Discussion

For the participating students, the seminars were able to help raise awareness of thought and decision-making processes, encouraging the students to critically examine and articulate them in words. The students in both learning environments felt themselves to be most familiar with scientific reasoning. The students studying at the masters level possessed more professional experience and in part held leadership positions, which could explain why they felt better able to use conditional, narrative and ethical reasoning and felt better qualified to engage in interactive reasoning than the undergraduate students. Systematic reasoning for those first beginning professional practice can lead to a lack of reflection on patterns of reasoning and can mean that reasoning processes are not adapted to specific situations [27]. CR enables a complex understanding of cases and encourages diagnostic performance during education if, along with systematic practice, other perspectives are integrated into specific decision-making processes [12], [13], [19], [28], [29]. Woods describes that for diagnostic decisions which are made with increasing professional experience, the knowledge gained from that experience along with intuition are increasingly drawn upon, while basic knowledge is pushed into the background. Diagnostic errors can also happen in these cases where there is no consistent inclusion of standard knowledge in the decision-making process [30], [31]. Focus can be placed specifically on the possibility of errors when teaching CR at more advanced levels of education [32], [33]. The differentiated development and fostering of the forms of reasoning during all educational phases, including further education could contribute to balancing and successfully synthesizing different sources of knowledge and decision-making processes.

## 5. Conclusion

Addressing CR helps biomedical scientists to understand the complexity of professional practice, to recognize the corresponding need for professionalism and formal qualifications, and to critically analyze one’s own decision-making processes. CR for biomedical scientists can contribute to making the decision-making process visible to other occupational groups and thus integrate professional expertise advantageously into the provision of health care.

Over the long term, an interdisciplinary focus on CR could foster and promote the development of a shared discourse. Consistent, longitudinal anchoring of appropriate learning content into the undergraduate and graduate curricula of all medical and health care professions would be necessary. As such, CR represents a very promising practical model for interprofessional education.

## Competing interests

The authors declare that they have no competing interests. 

## Figures and Tables

**Table 1:**
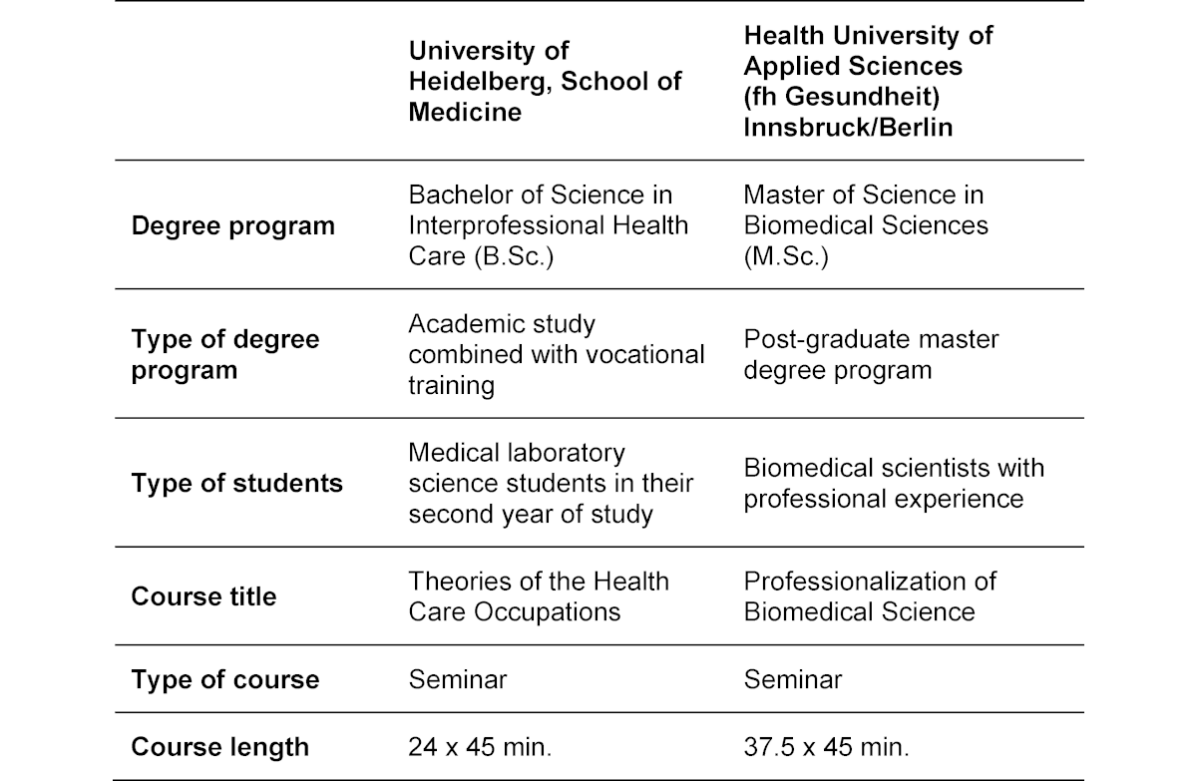
Clinical reasoning for biomedical science students in two educational settings

**Table 2:**
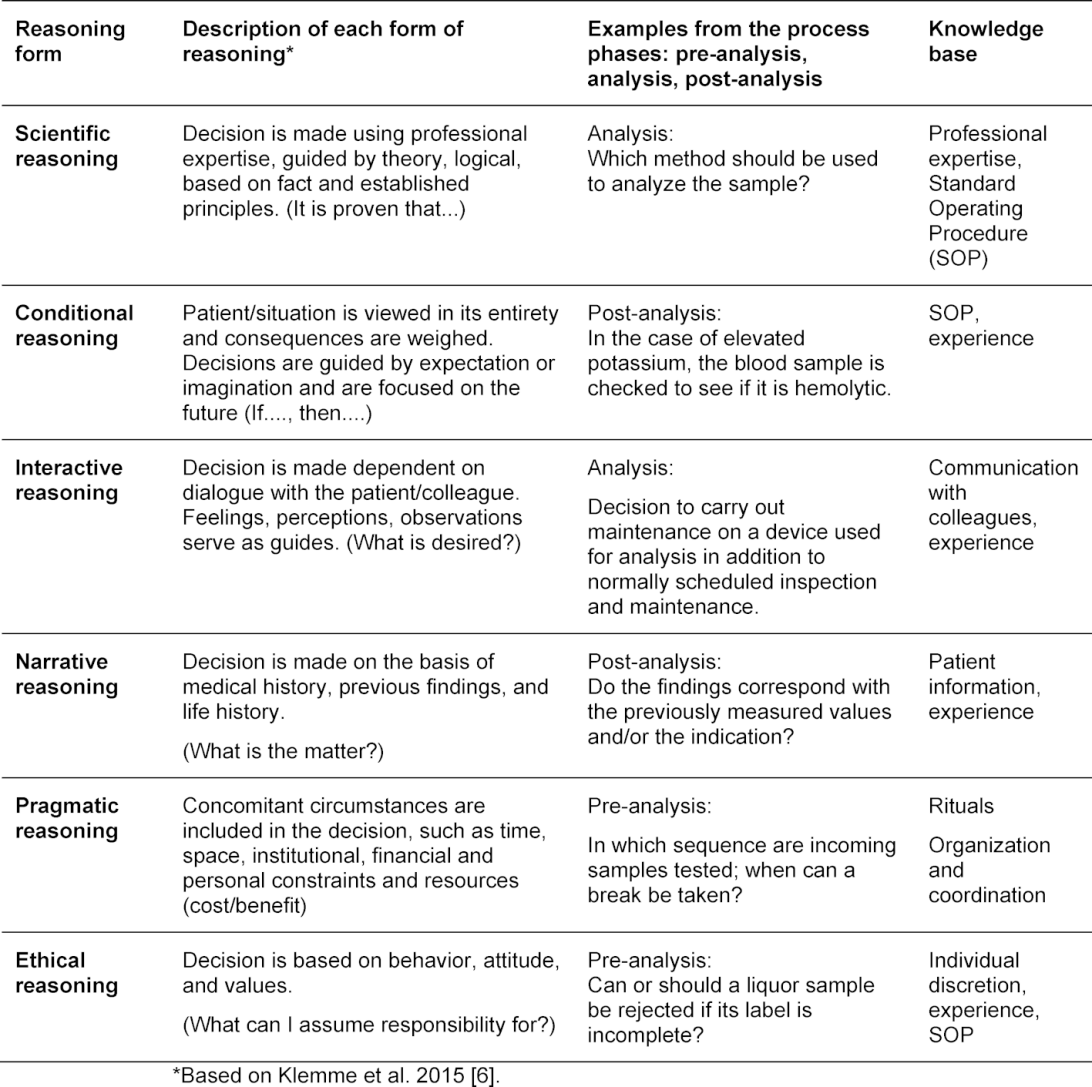
Forms of reasoning and practical examples (Heidelberg students)

**Table 3:**
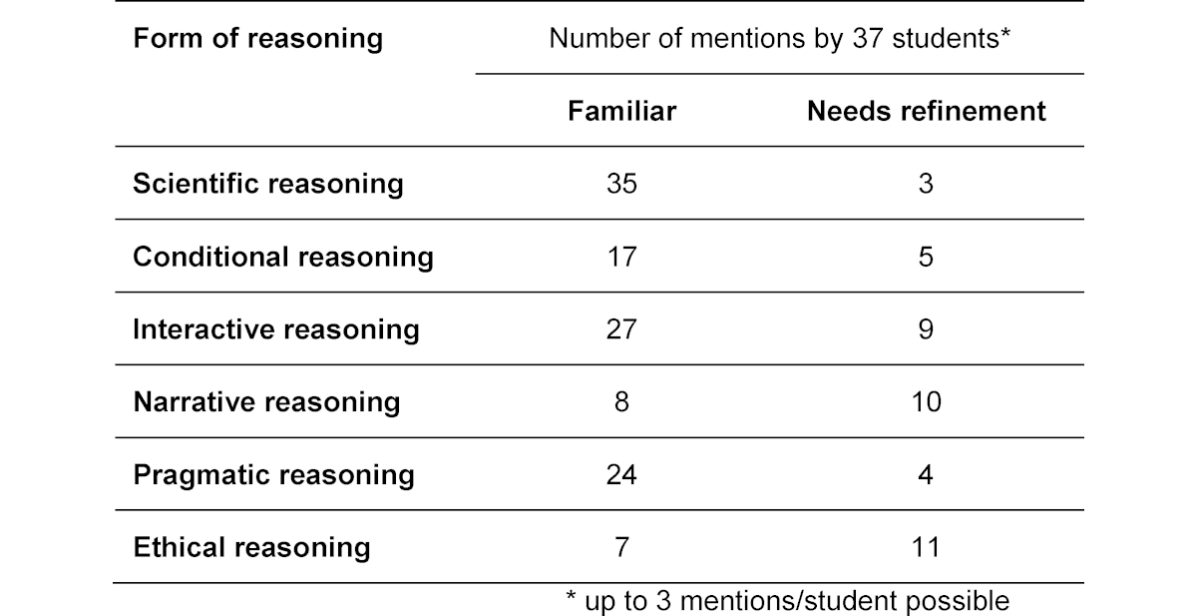
Familiar forms of reasoning and those needing refinement (Innsbruck/Berlin students)
